# Supplementary material for: Candidatus Desulforudis audaxviator dominates a 975 m deep groundwater community in central Sweden
Source: Commun Biol. 2024 Oct 15;7:1332. doi: 10.1038/s42003-024-07027-2 (PMC11480212; doi:10.1038/s42003-024-07027-2)
Supplement: Supplementary file 1 — Supplementary Information [file 42003_2024_7027_MOESM1_ESM.pdf]

Candidatus Desulforudis audaxviator dominates a 975 m deep groundwater community in central Sweden

George Westmeijer, Femke van Dam, Riikka Kietäväinen, Carolina González-Rosales,
Stefan Bertilsson, Henrik Drake, & Mark Dopson

Supplementary materials

Supplementary Table 1 Hydrochemistry. Samples were taken on 2 and 3 Sep 2022. Table is formatted as [conc pumped water] / [conc *in situ* sampling device]. Concentrations are shown in µg L^{-1}.

NO_3	Br^-	F^-	Cl^-	SO_4	Ag
0.024 / < 0.02	5.7×10^4 / 5.9×10^4	< 1.0	3.6×10^6 / 3.7×10^6	4.9×10^4 / 5.0×10^4	< 0.1
Al	Sb	As	Ba	Be	B
17	0.12 / 0.13	< 0.2 / 0.21	170 / 180	< 0.1	910 / 1000
P	I	Cd	K	Ca	Co
7.1 / 8.4	750 / 790	< 0.2	1.2×10^4	9.9×10^5 / 9.8×10^5	< 0.05
Cr	Cu	Li	Pb	Mg	Mn
< 0.2	< 0.1	2900	< 0.05	140 / 240	2.4 / 1.3
Mo	Na	Ni	Si	Fe	S
11 / 17	1.5×10^6	1.1	1.2×10^4 / 1.3×10^4	< 10	3.2×10^4 / 1.8×10^4
Rb	Se	Zn	Sr	Tl	Sn
88 / 85	< 0.2	< 1	1.7×10^4	< 0.1	< 0.2
Th	U	V	Bi		
< 0.05	< 0.01	< 0.05 / 0.072	< 0.05		

Supplementary Table 2 Sequencing details for metagenomic and amplicon data. The 16S and 18S ribosomal RNA gene amplicons and metagenomes were sequenced on an Illumina MiSeq (2 × 301 paired-end reads) and a NovaSeq with a SP flowcell (2 × 150 bp), respectively. The GTDB (release 207) was used for both 16S ASVs and reconstructed genomes while PR^2 (version 4.14) was used for annotating the 18S ASVs. The number of genomes refers to the number of unique de-replicated metagenome-assembled genomes.

**Type**	**Sample**	**DNA library (ng µL^{-1})**	**No. raw reads**	**No. ASVs / genomes**
	P28309_1061	8.40	91,004	177
16S rRNA	P28309_1062	3.37	51,909	134
	P28309_1063	6.23	91,356	218
16S rRNA control	P28309_1064	0.07	2,189	43
	WC-3642-S73	2.05	182,969	1,760
18S rRNA	WC-3642-S74	1.78	99,599	813
	WC-3642-S75	2.45	124,665	1,091
	VK-3516-S23	56.0	19.7 M	24
Metagenome	VK-3516-S30	99.6	25.2 M	26
	VK-3516-S31	73.0	16.1 M	25

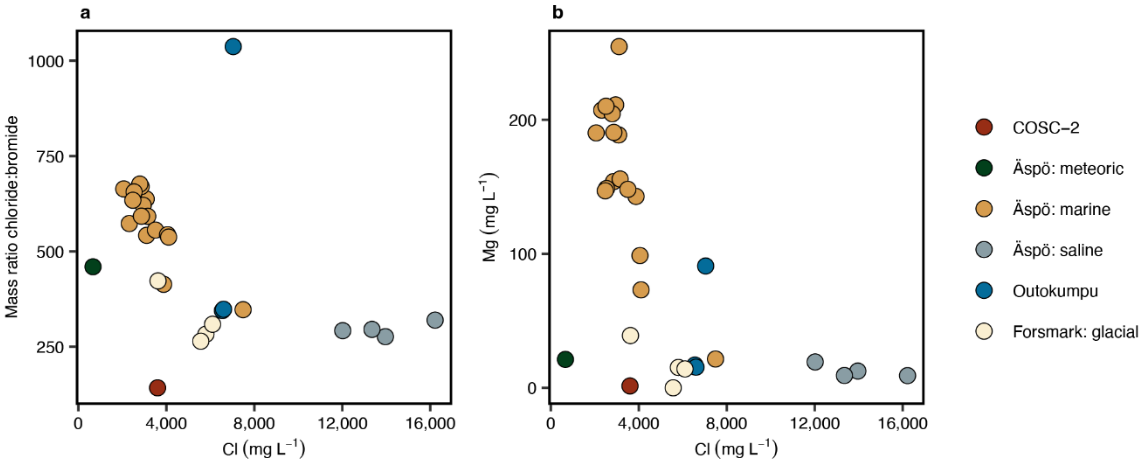

Supplemental Figure 1 Comparison of groundwater hydrochemistry. a) Chloride concentration (in mg L^{-1}) versus the mass ratio of chloride to bromide and **b)** Chloride concentration versus magnesium concentration (both in mg L^{-1}). The meteoric groundwater at Äspö Hard Rock Laboratory had a depth of 69 m below sea level while the Äspö marine and saline groundwaters were between 140 and 467 m depth. The glacial groundwaters at Forsmark were between 227 and 549 m depth (below sea level) and the Outokumpu borehole samples were taken from 500, 1000, and 1500 m depth.

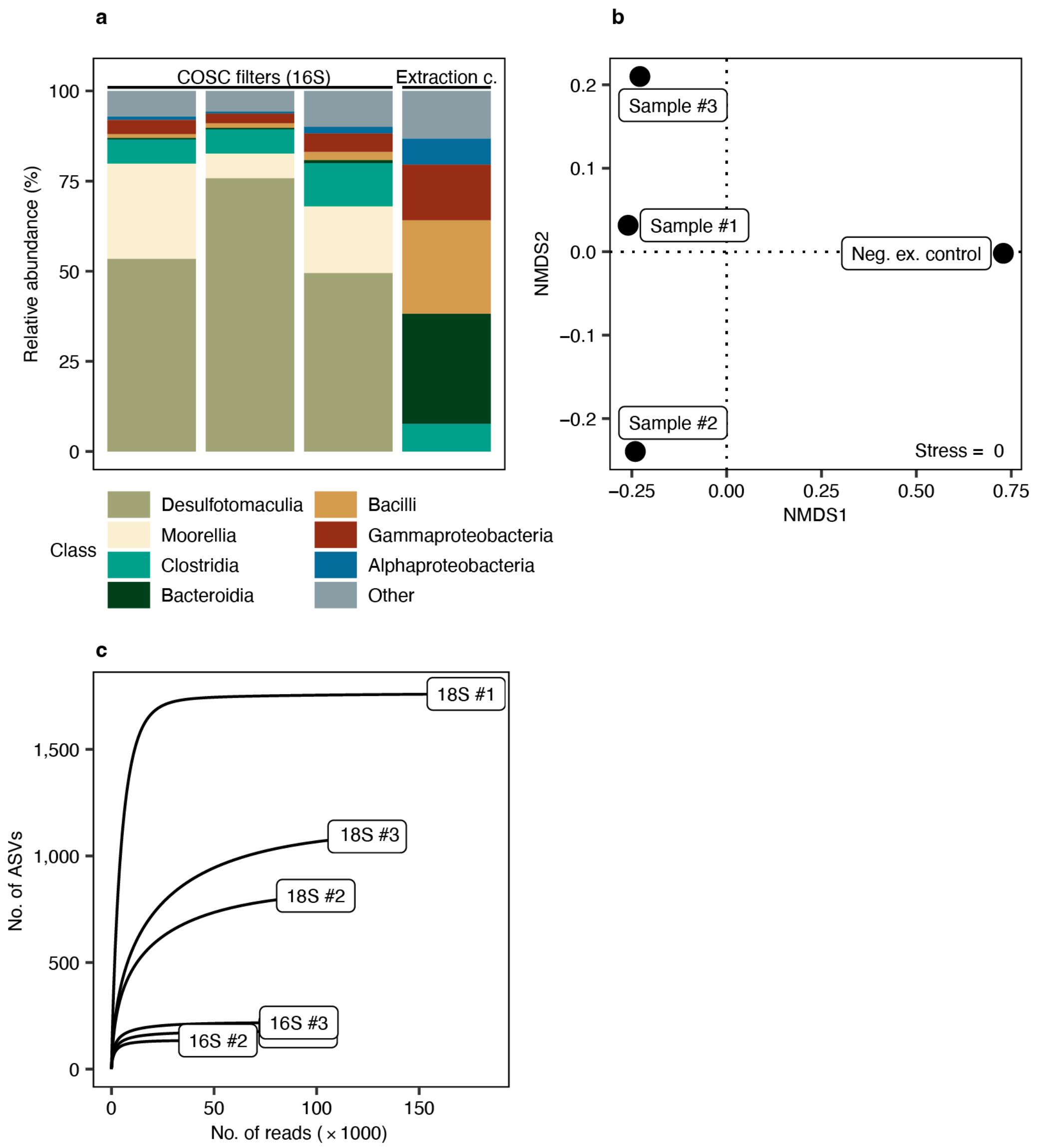

Supplemental Figure 2 Negative controls and rarefaction. a) Bacterial community composition depicted at the level of class. Classes are arranged according to abundance while grouping low-abundant taxa as "Other". **b)** Community divergence based on ASVs using non-metric dimensional scaling (NMDS). **c)** Rarefaction curves of both 16S and 18S ribosomal RNA gene amplicons.

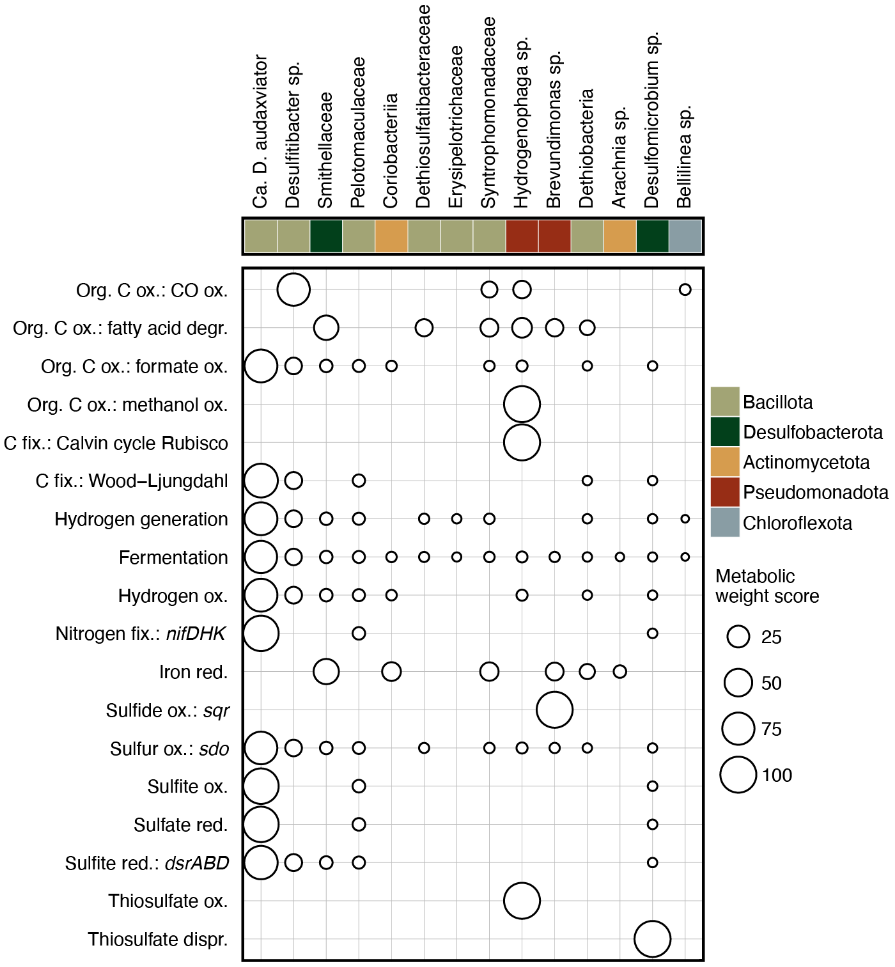

Supplemental Figure 3 Genomic potential according to metabolic weight score. The metabolic weight score (obtained using METABOLIC) is a measure of the metabolic potential combined with the read coverage (abundance) of the respective reconstructed genome in the community. The genomes are ordered according to descending coverage (left to right). The figure only shows genomes with a read coverage above 1 %. org; organic, ox; oxidation, degr; degradation, fix; fixation, red; reduction, dispr; disproportionation.
